# Effectiveness of the Promising Neighbourhoods community program in 0-to 12-year-olds**:** A difference-in-difference analysis

**DOI:** 10.1016/j.ssmph.2022.101166

**Published:** 2022-07-08

**Authors:** Mirte Boelens, Hein Raat, Harrie Jonkman, Clemens M.H. Hosman, Denis Wiering, Wilma Jansen

**Affiliations:** a Department of Public Health, Erasmus University Medical Center, Rotterdam, the Netherlands; b Verwey-Jonker Institute, Utrecht, the Netherlands; c Department of Health Promotion, Maastricht University, Maastricht, the Netherlands; d Department of Clinical Psychology, Radboud University, Nijmegen, the Netherlands; e Hosman Prevention and Innovation Consultancy, Berg en Dal, the Netherlands; f Department of Social Development, Municipality of Rotterdam, Rotterdam, the Netherlands

**Keywords:** SES, socioeconomic status, EPHE, EPODE for health equity, CTC, Communities that Care, NYI, Netherlands Youth Institute, SDQ, Strengths and Difficulties Questionnaire, NDC, England's New Deal for Communities

## Abstract

**Objective:**

The purpose of this study was to evaluate a collaborative community-based program that aims to a) increase the health, safety and talent development of youth, and b) contribute to the reduction of socioeconomic inequalities.

**Methods:**

A difference-in difference design with two separate cross-sectional samples in 2018 (n = 984) and 2021 (n = 413) among 0- to 12-year-olds with an intervention and comparator condition was used. The program, called Promising Neighbourhoods, consists of collaboration with community stakeholders, data-based priority setting, knowledge-and theory-based policies, and evidence-based interventions. The program was implemented in three neighbourhoods which were compared with three similar comparator neighbourhoods in which the program was not implemented. Logistic difference-in-difference regression was used to test effectiveness of the intervention on informal parenting support, outdoor-play, sport club membership, general health and risk of emotional and behavioural difficulties and to examine differences in intervention effects between children with a lower or higher socioeconomic status.

**Results:**

A significant intervention effect of the Promising Neighbourhoods program after two-years was found for outdoor-play (OR 0.61; 95%CI 0.37, 0.99). No other significant intervention effects were found for other outcomes. No different interventions effects were found for children with a lower or higher socioeconomic status on outcomes.

**Conclusion:**

The findings of this study indicate a positive intervention effect for one of the outcomes in 0- to 12-year-olds. Further mixed-methods evaluation research and using longer follow-up periods are needed to examine the value of these type of programs. Further development of Promising Neighbourhoods seems warranted.

**Trial registration:**

This study was prospectively registered in the Netherlands National Trial Register (Number: NL7279) on 26 September 2018.

## Highlights

• A collaborative neighbourhood program involving stakeholders was evaluated.
• The program included joint priority setting to promote child health/well-being.
• A positive intervention effect was found for one outcome in 0- to 12-year-olds.
• No reduction in socioeconomic inequalities was found in 0- to 12-year-olds.

## 1 Introduction

Socioeconomic status (SES) influences the development and health of youth (Arcaya et al., 2015; Braveman et al., 2011). Socioeconomic inequalities have been demonstrated in the youngest age groups and often continue in adult life (Cheng et al., 2015; Marmot and Bell, 2012, 2016). Therefore, investing in the reduction of socioeconomic inequalities in children is of utmost importance (Marmot & Bell, 2019). Although much research has been performed on the magnitude and causes of socioeconomic inequalities, relatively less is available on effective approaches to reduce them (Macintyre et al., 2020; Marmot & Bell, 2019).

The Ottawa Charter already mentioned in 1986 that community involvement and creating supportive conditions and environments could be strategies to reduce inequalities and to increase health and well-being of the community (Kickbusch, 2003; World Health Organization, 2009). Intersectoral collaboration, community participation, creating healthy settings, political commitment, funding and infrastructure, employing multiple strategies and actions at multiple levels and awareness of the socio-environmental context were found to be key to the effectiveness of health promotion programs. (Jackson et al., 2006; National Academies of Science Engineering, 2019) The important role local governments can play in reducing health inequalities has also been stressed by the World Health Organization (WHO) as they have a responsibility for the planning and delivery of services such as education, transport, housing and urban planning (World Health Organization, 2012). Moreover, local governments are often in a strong position to bring a wide variety of local actors or stakeholders around the table to stimulate action (World Health Organization, 2012).

Collaborative community-based programs typically comprise many of the key actions mentioned above. They employ multiple interventions, involve key-leaders and networks, and aim to strengthen the community (Merzel & D'Afflitti, 2003). Therefore, they can be regarded as promising in reducing socioeconomic inequalities in the healthy and safe development of youth (Merzel & D'Afflitti, 2003; Wallerstein & Duran, 2010; Fagan et al., 2018).

In the past years there has been an increasing focus on local integrated community-based programming approaches (Halfon et al., 2022). An example is the EPODE-program for the Promotion of Health Equity (EPHE) which focused on reducing socioeconomic inequalities (Borys et al., 2016; Mantziki et al., 2014). In this program, communities developed and implemented tailored lifestyle interventions to the needs of different socioeconomic groups (Borys et al., 2016; Mantziki et al., 2014). The EPHE-program was successful in changing behaviour of children with a lower SES and with a higher SES (Borys et al., 2016). Another example is the Communities that Care (CTC) program that aimed to reduce problem behaviour among children and adolescents and was implemented at the neighbourhood level. In this program, they formed community coalitions, performed a needs assessment and chose which interventions needed to be implemented. CTC reduced health-risk behaviour in the United States of America and Australia (Hawkins et al., 2014; Rowland et al., 2018; Toumbourou et al., 2019; Oesterle et al., 2014; Oesterle et al., 2018; Rhew et al., 2013). In the United Kingdom and the Netherlands, the results were less favourable (Brown et al., 2007; Brown et al., 2011; Jonkman et al., 2009; Steketee et al., 2013). Results of these programs indicate that, indeed, community engagement and tailored programs could be promising but more research is needed to increase the evidence-base.

The Promising Neighbourhoods collaborative community-based program partly builds on the experience of earlier methodologies such as CTC and the EPODE-program (Arthur et al., 2010; Borys et al., 2016). The program was developed by the municipality of Rotterdam with the aim to increase the health, safety and talent development of youth living in Rotterdam, and to contribute to the reduction of socioeconomic inequalities (Wiering, 2015) The Promising Neighbourhoods program consists of collaborating with community stakeholders, data-based priority setting, knowledge-and theory-based policies and evidence-based interventions (Boelens et al., 2019). The results of the effect evaluation of this program on health outcomes and on health inequalities in 0-to 12-year-olds are described in this paper.

### 1.1 Research questions

1: What is the effectiveness of the Promising Neighbourhoods program in 0- to 12-year-olds on health outcomes (informal parenting support, outdoor-play, sport club membership, general health and risk of emotional and behavioural difficulties)?
2: What is the effectiveness of the Promising Neighbourhoods program in 0-to 12-year-olds on reducing socioeconomic inequalities in these health outcomes?

### 1.2 Study hypothesis

We hypothesize that the Promising Neighbourhoods program leads to improved health outcomes and to reduced socioeconomic inequalities on these outcomes in intervention neighbourhoods compared to comparator neighbourhoods. As integrated interventions will be offered by multiple stakeholders to individual and groups of children and their parents but specific for each neighbourhood, we expect not only benefits at the individual level but also on the neighbourhood level.

## 2 Methods

### 2.1 Study design

The study design has been described elsewhere (Boelens et al., 2019). Briefly, this study utilizes a difference-in difference design with two separate cross-sectional samples with an intervention and comparator condition. Measurements took place before implementation (T0) between May–July 2018 (T0) and at after implementation (T1) between April–July 2021.

This study was prospectively registered in the Netherlands National Trial Register (Number: NL7279) on 26 September 2018.

### 2.2 Setting

The setting has been described in a protocol paper for this study (Boelens et al., 2019). Briefly, this study took place in Rotterdam, the second largest city of the Netherlands (650 thousand inhabitants in 2020 of which 14% is between 0 and 12 years of age (Municipality of Rotterdam, 2020). Rotterdam is a multicultural city as 52% of the inhabitants has a migrant background (Municipality of Rotterdam, 2020). Rotterdam is one of the poorest cities in the Netherlands (12.8% of its inhabitants) (Statistics Netherlands, 2021). The city of Rotterdam has a low SES score and the differences in SES between neighbourhoods is high (Statistics Netherlands, 2022). Rotterdam comprises 14 city area's including 42 different neighbourhoods.

**Fig. 1:**
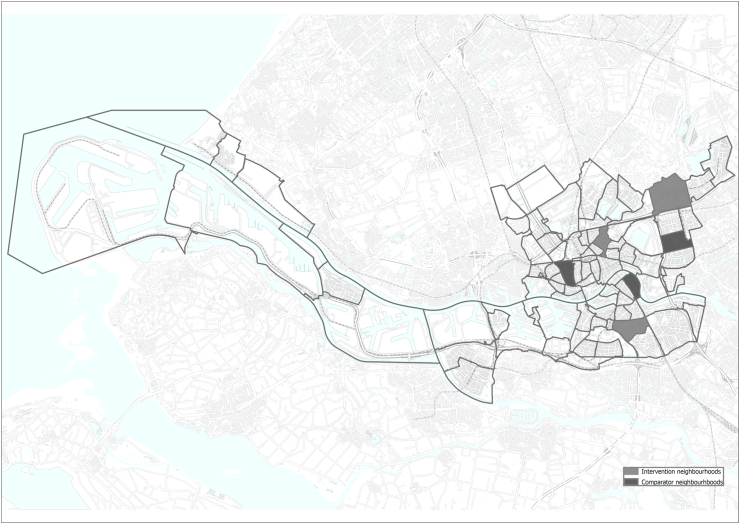
A geographical overview of intervention and comparator neighbourhoods in Rotterdam.

Neighbourhoods were defined administratively, based on their postal code. We did not use statistical techniques such as propensity score matching to select neighbourhoods. Rather, to ensure a sufficient diversity of SES and youth health problems in the neighbourhoods, we categorized neighbourhoods as low, middle or high degree of problems. The degree of problems is based on the percentage of low-educated inhabitants of <17 years-old, children with a non-Dutch migrant status, children aged 4 and 12 years old with a high score on the Strengths and Difficulties Questionnaire (SDQ) (e.g. emotional and behavioural difficulties) and the percentage of overweight children in grade two of primary school in the neighbourhoods. From each category an intervention neighbourhood was selected resulting in three intervention neighbourhoods. Similarly, from each category a comparator neighbourhood was chosen. Intervention and comparator neighbourhoods were comparable (See Supplemental Table 1). See Fig. 1 for a geographical overview of the intervention and comparator neighbourhoods. One comparator neighbourhood decided to implement common programming comparable to the Promising Neighbourhoods program on its own initiative. Therefore, one new comparator neighbourhood was chosen from the same category. The comparator neighbourhood that started with the common programming is used in the sensitivity analyses for comparison.

### 2.3 Study population

Data of 984 children aged 0-to 12-years old and were available at T0 and 413 at T1. Within these cross-sectional samples, respectively, 649 and 268 were 4-to 12-year-olds.

Invitations to participate to the cross-sectional surveys in 2018 and 2021 were done by drawing a random probability sample (representative for age and gender) from the municipal population register. Children living in a healthcare institution were excluded. Parents received invitations for one child only. All parents were living in Rotterdam when the survey was administered. At T0 the response rate for 0- to 4-year-olds was 40.6% (n = 6,771) and for 4-12-year olds the response rate was 37.7%(n = 10,029). At T1 the response rate for 0- to 4-year-olds was 37.0% (n = 160) and for 4- to 12-year-olds the response rate was 31.8% (n = 274). No information about characteristics of non-responders was available.

A power calculation to determine the sample size of the data needed to determine small effect sizes (f2 = 0.02) has been previously described (Boelens et al., 2019; Cohen, 2013). A sample of 818 (409 at T0 and 409 at T1) evenly distributed over the intervention and comparator neighbourhoods would provide sufficient power.

The sample at T0 was larger than that was needed to provide sufficient power and than the sample at T1. This was because we used data collected by the municipality for our T0 sample. This sample was also collected for other research and monitoring purposes.

### 2.4 The Promising Neighbourhoods program

The collaborative community-based program has been described extensively in the study protocol (Boelens et al., 2019). Briefly, the aims of the program are to increase the health, safety and talent development of youth (Wiering, 2015). The program is a collaborative community-based approach that includes community stakeholders, works with data-driven priority setting, knowledge- and theory-based policies and focuses on implementation of evidence-based interventions (Arthur et al., 2010; Borys et al., 2016). This program is continuously further developed and adjusted. Also during this study the program was further developed. The program is seen as a learning process.

The program is managed by municipal district advisors (Wiering et al., 2019). Each municipal district advisor is assigned to a different neighbourhood and coordinates and monitors the program. Together with community stakeholders and key-leaders from the neighbourhood network the municipal district advisor plans and develops a tailored intervention package for the neighbourhood. This package can consist of parenting support, preventive (health) interventions, youth welfare, preventive measures and activities to improve health, safety and talent development among youth.

The program consists of multiple steps (Wiering et al., 2019). Step 1 is a needs-assessment of the neighbourhood based on local quantitative registry and survey data. In step 2, the needs-assessment is discussed with the neighbourhood network to match the conclusions with qualitative insights based on their daily experiences and to gain local support by setting joint goals. In step 3, the needs-assessment is adapted based on the insights from step 2. Based on this assessment priorities for the neighbourhood are determined (data based-priority setting). Table 1 shows the priorities that have been set for the intervention neighbourhoods. In step 4, municipal district advisors and the neighbourhood network inventoried the current interventions, policy measures, actions and agreements in the neighbourhood and checked the evidence-base of the intervention in the database of Effective Youth Interventions of the NYI (https://www.nji.nl/nl/Databank/Databank-Effectieve-Jeugdinterventies). In step 5, the most appropriate and available interventions, policy measures, actions and agreements for the priorities are chosen by the municipal district advisors and neighbourhood network. A detailed neighbourhood intervention-package plan including the needs-assessment, priorities and policy measures, interventions and activities is developed in step 6. Table 1 shows the interventions, policy measures, actions and agreements that have been chosen in the intervention neighbourhoods for youth aged 0–18 year old. In step 7, this plan is implemented in the neighbourhood. Step 8 consists of continuous monitoring and evaluation.

**Table 1:** Overview of the priorities, interventions and policy measures, actions and agreements in intervention neighbourhoods for children aged 0- to 18-years old.

Priorities	Interventions	Policy measures, actions and agreements among partners
1) More children have better social-emotional health 2) Reduced risk of psychosocial problems 3) Reduced problem behaviour of the child 4) Fewer children are anxious 5) Fewer children are bullied 6) 50% of all children participate in a training for social emotional development as part of the school curriculum 7) More children are playing outside 8) An increase of children that participate in sports after school 9) More children have a better general health 10) More parents have informal parenting support 11) An increase in children that grow up in a save home environment 12) More young people perform better at school and obtain their school diploma 13) reduced relative school absence 14) The burden of crowd forming/hanging out on the streets among older youth is diminishing 15) A decrease in youth criminality	− 4 interventions for 0–4 year olds, mainly focused on parenting skills and socio- emotional skills − 10 interventions for primary school-aged children mainly focused on parenting skills, low SES and/or social emotional skills − 3 interventions focused on children of divorced parents − 4 interventions focused on children with parents who suffer from psychiatric problems or addiction − 7 interventions for youth from 12 to 25 mainly focused on socio-emotional skills − 16 interventions for parents mainly focused at parenting skills − 2 interventions focused on domestic violence and fights at home − 6 intervention only given at primary school focused at socio-emotional skills and resilience − 1 intervention only given at secondary school focused at socio-emotional skills and resilience − 2 intervention focused on delinquency and safety − 2 interventions focused on participation − 1 Intervention focused on poverty and debts Total = 57 interventions of which 3 interventions fell in two categories	1) Training teachers on social emotional development. 2) Improving collaboration and awareness and knowledge of preventive interventions on social emotional skills between schools and other partners. 3) Improving knowledge and awareness of parents and professionals about alcohol and drug use during pregnancy and parenthood. 4) Improve parenting skills, healthy lifestyles and reduce risk behaviour of children by providing more information to parents. 5) Implementing media classes as schools. 6) Actively promoting the pedagogical neighbourhood values at school and in the neighbourhood. 7) Square/playground programming on the various squares/playgrounds. 8) Improve early identification of conduct problems. 9) Focus on pregnant women and young families in collaboration with partners in neighbourhood. 10) Increase sport participation among primary school aged children by increasing the opportunities for sport through sport clinics, by increasing awareness on sport facilities, and by increasing accessibility. 11) Stimulate children to participate in sport, culture or side jobs using role models, by offering locations, offering work-learning trajectories, offering side jobs, and organizing activities for and with youth and training in language improvement for parents and children. 12) Reducing poverty and debt by increasing the reach of a debt-reduction program and by subsidies for sport and other activities using municipal funds. 13) Improved collaboration of schools with truant officers, police officers and social welfare teams to reduce school absenteeism and delinquency. 14) Good and sufficient homework guidance through, among other things, the use of community centers. 15) Improve collaboration between youth workers. 16) Expand collaboration among care and support professionals in the neighbourhood (general practitioners, physiotherapists, dietitians, etc) and schools. 17) Aligning the attention from neighbourhood network partners to language improvement. 18) Discuss approach for pupils that live in other neighbourhoods with higher problem levels.

The priorities differed between intervention neighbourhoods. Interventions, policy measures, actions and agreements differed between the intervention neighbourhoods.

The collaborative community-based program started in the summer of 2018 after the T0 measurement. This means the municipal district advisors started with step 1. We have the information regarding the selected interventions, policy measures, actions and agreements for the priorities that have been chosen. Insight in the actual implemented interventions is lacking. Consequently, we have no reliable information on reach of implemented interventions. The collaborative community-based program is still carried on. We did not study the costs of the program.

### 2.5 Comparator neighbourhoods

In the comparator neighbourhoods no collaborative community-based program (Promising Neighbourhoods program) was implemented. Interventions, policy measures and actions have occurred as usual. The city of Rotterdam provides resources for a selected group of interventions that could be provided at city or neighbourhood level. These interventions can include but are not limited to parenting support, socio-emotional skills training, and interventions for children of parents with a mental illness or addiction.

We have no indication that interventions were implemented in the comparator neighbourhoods above and beyond “usual practice” but this cannot be ruled out.

### 2.6 Data collection

The T0 measurement was derived from data gathered between May–July in 2018 using a Dutch public health survey administered by the municipal public health service in the city of Rotterdam. Data for the T1 measurement were gathered separately between April–July in 2021 using a similar survey and approach. Data collection took place in the same season. Data collection in 2018 and 2021 ended before the start of the summer vacation in this region of the Netherlands. Both surveys targeted parents/caretakers of 0- to 12-year-old children. Questionnaires were filled out by the main caregiver.

Parents received hardcopy invitation letters with information about the online survey and login details. Hardcopy questionnaires were sent with the first reminder. The questionnaires were available in Dutch, English and Turkish. Non-responders were contacted by telephone and were offered extra help in completing the questionnaire. Small incentives were used for both measurements.

### 2.7 Measures

The outcome measures were collected using the surveys at T0 (2018) and T1 (2021). Three outcome measures were measured in 0- to 12-year-olds (informal parenting support, outdoor-play and general health) and two in 4- to 12-year-olds (sport club membership and risk of emotional and behavioural difficulties. Subscales and subscale items reflecting the priorities that were set for the intervention neighbourhoods were additionally explored.

### 2.8 Outcome measures in 0- to 12-year-olds

#### 2.8.1 Informal parenting support

Informal parenting support was measured by the item: ‘Can you talk to your family, friends, acquaintances or neighbours about (problems with) raising your child?’ Answer categories were: Yes often, Yes regularly, Yes occasionally or No hardly ever or Never. This was dichotomized as ‘Yes’ (Yes often, Yes regularly) and ‘No’ (Yes occasionally, No hardly ever, Never). The first category was used as reference.

#### 2.8.2 Outdoor-play

Outdoor-play was measured by two items. The first item was: ‘On how many days per week does your child play outdoors?” Answer categories were: My child did not play outdoors last week, but would usually do that in an ordinary week, Never, 1, 2, 3, 4, 5, 6 or Every day. The second item assessed the time their child usually spends playing outdoors. Answer categories were: Less than half an hour per day, Half an hour to an hour per day, 1–2 h per day, 2–3 h per day or 3 h per day or longer. For both questions we asked parents to base their answer on the past week. We dichotomized these questions to: ‘Outdoor-play for ≥60 min for ≥5 days a week’ or ‘No’. The first category was used as reference.

#### 2.8.3 General health

General health was measured by the item ‘How would you describe your child's general health’ (Very good, Good, Alright, Not very good or Poor); this was dichotomized as ‘Good’ (Very good, Good, Alright) or ‘Poor’. The first category was used as reference.

### 2.9 Outcome measures in 4- to 12-year-olds

#### 2.9.1 Sport club membership

Sport club membership was measured by the item ‘How many days per week does your child sports with a club’. Parents were asked to base their answer on the past week. Answer categories were: Never, My child did not do any sports last week, but would usually do that in an ordinary week, 1, 2, 3, 4, 5, 6, or Every day. This was dichotomized as ‘Sports at a sport club for ≥1 day a week’ or ‘No’. The first category was used as the reference.

#### 2.9.2 Risk of emotional and behavioural difficulties

Risk of emotional and behavioural difficulties was measured using the SDQ (Strengths and Difficulties Questionnaire) which was embedded in the surveys. This is a validated questionnaire to measure risk of emotional and behavioural difficulties and consists of five subscales: emotional problems, conduct problems, hyperactivity, peer-problems and prosocial behaviour (Goodman, 2001; Goodman & Goodman, 2009; Theunissen et al., 2016). We calculated the total difficulties score by adding the scores of all domains except for prosocial behaviour (Cronbach's alpha = 0.75). We dichotomized the total difficulties score using age dependent cut-offs to either ‘Normal score’ or ‘High risk’ with the normal score as reference category. For 4- to 7-year-olds a cut-off of ≥15 and for 7- to12-year-olds a cut-off of ≥14 indicates risk of emotional and behavioural difficulties (Goodman, 2001; Goodman & Goodman, 2009; Theunissen et al., 2016). We used the SDQ guidance and Dutch cut-offs for computing the scores and categorized outcomes.

Several subscales and scale items were additionally explored. The subscale emotional problems (Cronbach's alpha = 0.67) consists of five items about somatic symptoms, worries, feeling unhappy, being nervous in new situations and being anxious. The subscale conduct problems (Cronbach's alpha = 0.50) consists of five items about tantrums, obeying, bullying, lying, and stealing. Answer categories were: Not true, Somewhat true, or Certainly true. We computed subscale scores by adding the scores of all five items. We dichotomized these scores using age dependent cut-offs to either ‘Normal score’ or ‘High risk’ with the normal score as reference category. A score of 4–10 indicates emotional problems. A score of 3–10 indicates conduct problems (Goodman, 2001; Goodman & Goodman, 2009; Theunissen et al., 2016). We also used the following individual items: anxiety from the subscale emotional problems, tantrums, bullying, and stealing from the subscale conduct problems and being bullied from the peer-problem subscale. These were dichotomized as ‘No’(Not true) or ‘Yes’ (Somewhat true, Certainly true) with the first category as reference.

### 2.10 Covariates

#### 2.10.1 Sociodemographic measures

Age was measured continuously in years. Gender was measured as *‘Boys’* or ‘*Girls’* using the first as reference category.

#### 2.10.2 Socioeconomic status (SES)

Parental educational level was used as indication of SES and was defined as highest parental educational level obtained and categorized as *‘Higher’* (Higher vocational training, University degree, or Higher) or *‘Lower and intermediate’* (No education, Primary school, ≤4 years general secondary school, >4 years general secondary school or Intermediate vocational training). For the categorization we used the Dutch standard classification of education 2016 which is ISCED-F 2013 (Statistics Netherlands, 2016). The first category was the reference category.

### 2.11 Statistical analysis

Participant characteristics and health outcomes were described at T0 and at T1 for the intervention and comparator neighbourhoods. Differences were tested using chi-square or Mann-Whitney U tests (p < 0.05).

Multiple imputation (m = 5) using a fully conditional specified model (iterative Markov chain Monte Carlo (MCMC) method) based on the relationships between the variables included in this study was used for missing values. Multiple imputation was performed for variables measured for 0-12-year olds (2.1% missing values) and for variables measured for 4-12-year olds (0.6% missing values). We used 5 imputations as the amount of missing values was quite low.

Logistic difference-in-difference regression analysis was used to test intervention effects for the outcomes as well as differences in intervention effects according to SES (parental education). Difference-in-difference regression is a useful technique when randomization on the individual level is not possible. Difference-in-difference regression requires data from pre-/post-program implementation, such as repeated cross-sectional data. The approach removes biases in post-intervention period comparisons between the intervention a group (i.e. neighbourhoods) and comparator group (i.e. neighbourhoods) that could be the result from permanent differences between those groups, as well as biases from comparisons over time in the intervention group (i.e. neighbourhoods) that could be the result of trends due to other causes of the outcome. For the difference-in-difference regression analyses we computed two models. We also visualized the historical time trend assumption using data from 2014 on all outcome variables besides informal parenting support, that was rephrased in the 2018 version. In 2014 a similar Public Health survey as in 2018 was administered by the municipality of Rotterdam. The outcomes in 2014, 2018 and 2021 were plotted in a scatterplot in excel (See Supplemental Figs. S1–4). Trends between 2014 and 2018 seem comparable for intervention and comparator neighbourhoods.

In the first model we examined the intervention effect. The coefficient β3 of the interaction term between the condition (intervention or comparator) and time of measurement (T0 or T1) depicts the intervention effect on the outcome. We adjusted for SES (parental education), gender and age. This model can be written as:

γ=β0+β1*timeofmeasurement+β2*condition+β3*timeofmeasurement*condition+C(SES,genderandage)

The second model examined if the intervention effect differed according to SES (parental education). A three-way interaction between time of measurement, condition and SES (parental education) was added and all possible underlying two-way interactions. We also adjusted for age and gender. In this model β7 is the key-parameter. This model can be written as:

γ=β0+β1*timeofmeasurement+β2*condition+β3*SES+β4*timeofmeasurement*condition+β5*timeofmeasurement*SES+β6*condition*SES+B7*timeofmeasurement*condition*SES+C(age,gender)

Pooled effect estimates (odds ratios [ORs] and 95% confidence intervals (CIs) from these five datasets were reported. Two-sided p-values denoted statistical significance (p < 0.05).

Interaction effects between age and the condition were tested (age was dichotomized as 0–9 and 10–12 or 4-9 and 10–12 depending on the outcome variable) in model 2 to check for any differences due to age and none were found (p > 0.05). No further analysis by subgroups of age was needed. We also examined the impact of COVID-19. We checked whether there were significant differences between 2018 and 2021 on the outcome variables and covariates. Further we examined whether parents indicated that they attended less interventions/activities in their neighbourhood due to COVID-19.

Exploratory analyses were performed in the multiple imputed data in a similar way as the main analyses (model 2 and model 3). We repeated our analyses using a complete-case dataset (i.e. without missing values on outcome variables or covariates in children aged 0- to 12-year-olds and children aged 4- to 12-year-olds). As one comparator neighbourhood was replaced at the beginning of the study, we did a sensitivity analysis with the replaced neighbourhood instead of the new comparator neighbourhood. Further, we examined the distribution of outcomes and covariates across SES (parental education) between intervention and comparator neighbourhoods over time.

IBM SPSS statistics for Windows, version 25.0 (International Business Machines Corporation, Armonk, New York) was used for all analyses.

## 3 Results

Table 2 shows the characteristics of the study population (Supplemental Table 2 includes missing values and Supplemental Table 3 includes p-values for changes between comparator neighbourhoods at T0 and T1 and for intervention neighbourhoods at T0 and T1). At T0, children in comparator neighbourhoods were on average older than children in intervention neighbourhoods. Further no significant differences at T0 were found. This indicates sufficient comparability between intervention and comparator neighbourhoods. Over time, significantly more parents received informal parenting support in both comparator and intervention neighbourhoods. Outdoor-play significantly reduced over time in comparator neighbourhoods and increased in intervention neighbourhoods.

**Table 2:** Characteristics of the intervention and comparator neighbourhoods at baseline in 2018 and in 2021.

	2018 (n = 984)			2021 (n = 413)		
	Comparator neighbourhoods (n = 427; 43.4%)	Intervention neighbourhoods (n = 557; 56.6%)	p-value for differences at T0	Comparator neighbourhoods (n = 170; 41.2%)	Intervention neighbourhoods (n = 243; 58.8%)	p-value for differences at T1
**Socio-demographic variables**						
Age, continuous	6.0 (3.0–9.0)	5.0 (2.0 (8.0)	**0.034**	6.0 (2.8–9.0)	5.0 (2.0–9.0)	0.748
Age, dichotomous			**0.027**			0.930
*0–4*	132 (30.9%)	210 (37.7%)		59 (34.7%)	85 (35.1%)	
*4–12*	295 (69.1%)	347 (62.3%)		111 (65.3%)	157 (64.9%)	
Age, categories			0.062			0.670
*0–4*	132 (30.9%)	210 (37.7%)		59 (34.7%)	85 (35.1%)	
*4–10*	224 (52.5%)	254 (45.6%)		78 (45.9%)	118 (48.8%)	
*10–12*	71 (16.6%)	93 (16.7%)		33 (19.4%)	39 (16.1%)	
Gender			0.748			0.969
*Boy*	216 (50.6%)	276 (49.6%)		81 (47.9%)	116 (47.7%)	
*Girl*	211 (49.4%)	281 (50.4%)		88 (52.1%)	127 (52.3%)	
**SES**			0.188			0.251
Parental education						
*Higher*	228 (55.6%)	276 (51.3%)		104 (61.9%)	134 (56.3%)	
*Lower and intermediate*	182 (44.4%)	262 (48.7%)		64 (38.1%)	104 (43.7%)	
**Outcomes in 0- to -12-year-olds**						
Informal parenting support			0.108			0.855
*Yes*	238 (56.3%)	340 (61.4%)		118 (69.8%)	171 (70.7%)	
*No*	185 (43.7%)	214 (38.6%)		51 (30.2%)	71 (29.3%)	
Outdoor-play			0.281			**0.004**
*Yes*	168 (41.6%)	232 (45.1%)		49 (31.0%)	103 (45.8%)	
*No*	236 (58.4%)	282 (54.9%)		109 (69.0%)	122 (54.2%)	
General health			0.815			0.282
*Good*	389 (92.0%)	509 (91.5%)		163 (95.9%)	227 (93.4%)	
*Not good*	34 (8.0%)	47 (8.5%)		7 (4.1%)	16 (6.6%)	
**Outcomes in 4- to- 12-year-olds**						
Sport club membership			0.764			0.228
*Yes*	164 (56.7%)	190 (55.6%)		65 (59.1%)	80 (51.6%)	
*No*	125 (43.3%)	152 (44.4%)		45 (40.9%)	75 (48.4%)	
Risk of emotional and behavioural difficulties			0.967			0.142
*No*	257 (88.9%)	302 (88.8%)		102 (92.7%)	135 (87.1%)	
*Yes*	32 (11.1%)	38 (11.2%)		9 (7.3%)	20 (12.9%)	

P-values computed using chi-square for categorical variables and Mann-Whitney U tests for continuous variables.

**Bold** indicates a significant difference between intervention and comparator neighbourhoods (i.e. p < 0.05).

Table 3 shows the main results. There is an intervention effect of the Promising Neighbourhoods program on outdoor-play. No other intervention effects on the outcomes were found (Model 1). There were no significant different intervention effects for children with a lower or higher SES on the outcomes.

**Table 3:** Logistic difference-in-difference regression analyses.

	Informal parenting support 0- to 12-year-olds	Outdoor-play 0- to 12-year-olds	General health 0- to 12-year-olds	Sport club membership 4- to 12-year-olds	Risk of emotional and behavioural difficulties 4- to 12-year-olds
	OR (95% CI) for N = 1,397	OR (95% CI) for N = 1,397	OR (95% CI) for N = 1,397	OR (95%CI) for N = 896	OR (95%CI) for N = 896
Model 1	Two-way interaction parameter estimates (intervention condition in 2021)				
	1.23 (0.66, 2.26)	**0.61 (0.37, 0.99)**	1.55 (0.56, 4.34)	1.18 (0.71, 1.97)	1.95 (0.72, 5.33)
Model 2	Three-way interaction parameter estimates (difference in inequalities for the intervention condition in 2021)				
	0.59 (0.16, 2.09)	0.96 (0.34, 2.68)	0.82 (0.10, 6.57)	0.41 (0.14, 1.16)	0.95 (0.11, 8.05)

An odds ratio <1.00 indicates a favourable change in the outcome. **Bold** indicates statistical significance p < 0.05. Model 1 is adjusted for age (continuous), gender (ref = boy) and parental education (ref = high), and includes a two-way interaction of time of measurement*condition.

Model 2 is adjusted for age (continuous) gender (ref = boy) and parental education (ref = high) and includes two-way interactions of time of measurement*condition, time of measurement*parental education, condition*parental education and a three-way interaction of time of measurement*condition*parental education.

The impact of COVID-19 was examined in the T1 measurement. Of all parents, 37.5% responded that there were less interventions/activities in their neighbourhood due to COVID-19. There was no significant difference between intervention and comparator neighbourhoods. We compared the T0 and T1 measurements in the whole sample and found that in general outcomes were similar in 2018 and 2021 (Supplemental Table 2). Only the percentages of parents with informal parenting support was significantly higher at T1 in 2021 compared to the T0 measurement in 2018.

Supplemental Table 4 shows the exploratory analyses. No significant intervention effects were found for subscales or items of the SDQ. There were also no significant different intervention effects for children with a lower versus a higher SES.

The complete-case analyses were similar to the main analyses except that for informal parenting support a significant different intervention effect was found for children with a lower or higher SES (Supplemental Table 5). This might be an incidental finding. As sensitivity analysis we repeated the analyses with the originally included comparator neighbourhood that started with common programming during this study instead of the alternative comparator neighbourhood that was chosen later on (Supplemental Table 6). These analyses were similar to the main analyses. The distribution across SES between intervention and comparator neighbourhoods over time is shown in Supplemental Table 7. This table shows that the percentage of parents of 0- to 12-year-olds with good informal parenting support among low SES groups shows a higher increase over the years compared to the high SES groups. For outdoor-play it shows that the percentage of 0- to-4-year-olds in comparator neighbourhoods decreased irrespective of SES and increased in intervention neighbourhoods.

## 4 Discussion

We examined the effectiveness of the Promising Neighbourhoods program in 0- to-12-year-olds on different outcomes. We found a positive intervention effect on outdoor-play. We found no other significant intervention effects and no differential effects for children with a lower or higher SES on the outcomes.

We found a positive intervention effect on the outcome outdoor-play. Merzel and D'Afflitti conducted a review on community programs and found that, in general, programs employing community networks have a limited impact on population health (Merzel & D'Afflitti, 2003). The authors reported that the modest impact is a result of multiple factors such as methodological limitations and a limited scope and intensity (Merzel & D'Afflitti, 2003). To illustrate, a modest impact has also been found in a study evaluating the impact of the New Deal for Communities Program (NDC) in England (Lawless & Pearson, 2012). The NDC was an urban regeneration program employing collaborative community engagement in 39 areas on different topics such as crime, housing and health which was compared to comparator areas with similar deprivation. Despite this, collaborative community-based programs such as Better Start Bradford are seen as promising for reducing inequalities in child health (Halfon et al., 2022).

There are multiple explanations for finding an intervention effect on only one outcome (outdoor-play) of the Promising Neighbourhoods program in 0- to12-year-olds (Merzel & D'Afflitti, 2003). First, a possible explanation for finding a positive intervention effect on outdoor-play only could be the implementation of interventions in comparator neighbourhoods. This is one of the reasons proposed by Merzel and D'Afflititi (Merzel & D'Afflitti, 2003). Additionally, gathered process indicators show that 24.0% of children/parents from intervention neighbourhoods and 25.9% in comparator neighbourhoods participated in interventions (e.g. preventing bullying, overcoming fear, language and learning, mental health, social skills, outdoor-play, healthy food and exercise or other) in the past year at T1. These percentages did not differ significantly. We are unaware if interventions implemented in comparator neighbourhoods fell under care-as-usual or if additional interventions were implemented.

Another explanation might be that the Promising Neighbourhoods program needs to be implemented for a longer period of time before more intervention effects can be expected. Our follow-up measurement took place two years after the start of the program aligned with the planning of the Promising Neighbourhoods program. An additional follow-up measurement after a longer follow-up period is warranted to give insights in possible intervention effects after a prolonged implementation period. In another community-based program called “Arnhemse Broek, Healthy and Well” which was implemented in 2004 in neighbourhoods in a Dutch city (Arnhem) a follow-up period of two year also seemed to short (Abbema et al., 2004). This community-based program that targeted adults followed a similar approach; locally active professionals of varying organizations (e.g. police or youth work) formulated an action plan which included priorities, activities and actions for themes such as parenting problems and social safety. In their effect evaluation after two years, some positive effects were found but more negative effects were reported (Abbema et al., 2004). Unfortunately, the NDC program was implemented for ten years and not many effective results were reported (Lawless & Pearson, 2012).

Another explanation might be that some of the chosen interventions were not the most effective interventions to achieve favourable changes on the priorities chosen. If so, this could have impacted the effectiveness of the Promising Neighbourhoods program. The municipal district advisors and local stakeholders select interventions they view as most appropriate and suitable for their local community. In the Promising Neighbourhoods program the priorities were chosen after the T0 measurement took place and not beforehand. The T0 survey measured health indicators that were deemed relevant for local health and youth policies. However, some priorities that were chosen in the intervention neighbourhoods were not measured with the survey.

Finally, it could be that the Promising Neighbourhoods program was not delivered as intended beforehand (Mitchell et al., 2002; Stith et al., 2006). However, the likelihood of demonstrating population level change of collaborative community-based programs can be challenging because of the complexity, context and specific features that make it difficult to use traditional evaluation methods (Lifsey et al., 2015). Contextual factor associated with the effectiveness can differ between communities (Lifsey et al., 2015). A thorough process evaluation, taking into account the logic model that was set up for this evaluation, will shed more light on the implementation and increase our understanding of barriers and facilitators for the implementation of community-based programs (Dickerson et al., 2019; Evans et al., 2015).

## 5 Methodological considerations

During the implementation of the Promising Neighbourhoods program in 2019 COVID-19 became a global pandemic. Due to the COVID-19 pandemic, interventions were cancelled, postponed or continued as online intervention. COVID-19 could have influenced the outcome measures in both intervention and comparator neighbourhoods but it may be possible that the effects turned out differently in intervention and comparator neighbourhoods. Moreover, our results regarding the effectiveness of the Promising Neighbourhoods program may not be generalizable to a situation without COVID-19.

Our study has several strengths. For the current analysis we not only studied the effect of the Promising Neighbourhoods program in intervention neighbourhoods compared to comparator neighbourhoods but also whether differences between children with a lower and a higher SES reduced as a consequence of the program. We used a difference-in-difference approach, which is a suitable technique to study effects of such community-based programs. Risk of emotional and behavioural difficulties was measured using the SDQ, which is a validated questionnaire (Goodman, 2001; Goodman & Goodman, 2009; Theunissen et al., 2016). We conducted several additional analyses that are similar to our main findings.

Several limitations of our study need to be taken into consideration when interpreting the findings. First, contamination between the intervention and comparator neighbourhoods could have occurred. For example, when parents and their children moved from an intervention to a comparator neighbourhood or vice versa. Children and/or parents from comparator neighbourhoods could also attend schools in the intervention neighbourhoods and benefit from implemented interventions. Some intervention and comparator neighbourhoods are in close proximity of each other (See also Fig. 1). This could have influenced the findings of our evaluation. We unfortunately do not have data to check whether this could have been the case. Second, it could be that there were interventions implemented in the comparator neighbourhoods. This could lead to null findings but is inherent to the design of a collaborative community-based program in the real world. For example, community stakeholders in one comparator neighbourhood started themselves with common programming. We have performed the analyses also using this comparator neighbourhood. However, the results were similar. Third, we used parental education as an indicator of SES in our analyses. Other indicators of SES might have yielded different results. The risk of a low income is the highest if the main breadwinner of the family attained lower education. Rotterdam is the city with the highest percentage of households living in poverty in the Netherlands (Statistics Netherlands, 2021). Rotterdam is a city with relatively lower educated inhabitants (i.e. lower 31%, middle 38%, and higher 31%) compared to the average in the Netherlands (i.e. lower 28%, middle 42% and higher 31%) (Municipality of Rotterdam, 2021). This indicates that educational level is related to a low income. However, it could be that we miss SES differences by only looking at differences between lower and higher educated participants. Fourth, the sample size for 4-to 12-year-olds at follow-up was somewhat lower than needed to detect small effect sizes. Fifth, only the SDQ was a validated measure. We cannot be sure that the other outcome measures (i.e. informal parenting support, outdoor-play, general health, sport club membership) measure what they intend to measure or if they are able to measure change (or change across SES). We selected these outcome measures because they matched the priorities that were chosen. For some of the priorities no suitable outcome measure (i.e. youth criminality) was present. Finally, this study took place in neighbourhoods of a large Dutch city. Findings may not be generalizable to other settings such as neighbourhoods in smaller cities, rural areas or other countries.

### 5.1 Future research

For the evaluation of Promising Neighbourhoods and comparable programs more follow up measurements or a longer follow-up period is warranted as intervention effects might need a longer implementation period. The effectiveness in older youth still needs to be evaluated.

Several key actions for successful health promotion programs have been reported by Jackson et al. (2006). Collaborative community-based programs like Promising Neighbourhoods include many of these key actions in their design. Perhaps, these key-actions currently were not or not yet adequately incorporated or not sufficiently addressed in the Promising Neighbourhoods program for the program to be effective. Key actions such as intersectoral collaboration and interorganizational partnerships or community participation might just need more time to establish and become effective. It is also possible that there are other key actions needed for effective collaborative community-based programs that have not yet been identified in the study by Jackson et al. (2006). Future research to community-based programs is warranted to provide these necessary insights.

Further, by additionally studying the implementation process of such programs from other perspectives such as interviews with policymakers or content analyses of policy documents will provide more insights in underlying mechanisms. Taking into account the context and how children/families experience the implementation of collaborative community-based programs like Promising Neighbourhoods could also provide relevant insights.

Information on the cost-effectiveness of the Promising Neighbourhoods program and similar programs would further inform local health promotion policies and needs further research.

## 6 Conclusion

The findings of this study indicate a positive intervention effect for one of the outcomes in 0- to 12-year-olds. Further mixed-methods evaluation research and using longer periods between measurements are needed to examine the value of these type of programs. Further development of Promising Neighbourhoods seems warranted.

## Ethics

The medical ethics committee of the Erasmus University Medical Center Rotterdam declared the Medical Research Involving Human Subjects Act does not apply and issued a declaration of no objection for this study (MEC-2018-1506). Parents received information about the study and could refuse participation by not filling out the survey.

## Declaration of competing interest

The authors have no conflict of interest relevant to this article to disclose.

## Funding/support

This work was funded by a research grant (project number: 531001313) from 10.13039/501100001826ZonMw, The Netherlands Organization for Health Research and Development.

## Role of funder/sponsor

ZonMw has no role in any part of the research, writing and reviewing of the manuscript.

## Availability of data and materials

Data used for the T0 measurement in 2018 were obtained from a Dutch Public Health survey carried out in 2018 by the municipal public health service in the city of Rotterdam (Gezondheidsmonitor Kinderen GGD Rotterdam-Rijnmond). The data are protected by the Municipal Health Service of Rotterdam. Data are available under request via: gezondheidsmonitorbco@rotterdam.nl. Data used for the T1 measurement is available upon request from the corresponding author.

## CRediT authorship contribution statement

**Mirte Boelens:** Conceptualization, Methodology, Formal analysis, Investigation, Writing – original draft. **Hein Raat:** Conceptualization, Writing – review & editing, Supervision, Funding acquisition. **Harrie Jonkman:** Writing – review & editing, Funding acquisition. **Clemens M.H. Hosman:** Writing – review & editing, Funding acquisition. **Denis Wiering:** Writing – review & editing. **Wilma Jansen:** Conceptualization, Writing – review & editing, Supervision, Funding acquisition.
