# Supplementary material for: Effectiveness of the Promising Neighbourhoods community program in 0-to 12-year-olds: A difference-in-difference analysis
Source: SSM Popul Health. 2022 Jul 8;19:101166. doi: 10.1016/j.ssmph.2022.101166 (PMC9289725; doi:10.1016/j.ssmph.2022.101166)
Supplement: Multimedia component 1 [file mmc1.docx]

**Figure S1.** Historical trend assumption for sufficient outdoor play (≥5 days per week 1 hour).

**Figure S2.** Historical time trend for general health (good).

**Figure S3.** Historical time trend for sport club membership (member of a sport club and sports there at least 1 hour per week).

**Figure S4.** Historical time trend for risk of emotional and behavioural difficulties (not-at-risk score).
