# Supplementary material for: Effectiveness of the Promising Neighbourhoods community program in 0-to 12-year-olds: A difference-in-difference analysis
Source: SSM Popul Health. 2022 Jul 8;19:101166. doi: 10.1016/j.ssmph.2022.101166 (PMC9289725; doi:10.1016/j.ssmph.2022.101166)
Supplement: Multimedia component 2 [file mmc2.docx]

| **Supplemental table 1.** balance between intervention and comparator neighbourhoods at T0 in 2018. | | | | |
| --- | --- | --- | --- | --- |
| Neighbourhoods | Percentage low educated <17-year-olds | Percentage children with a non-Dutch migrant status | Percentage of 4 to -12-year-old children at risk of emotional of behavioural difficulties | Percentage of children with overweight in grade 2 |
| Low degree of problems |  |  |  |  |
| *Intervention* | 8 | 32 | 8 | 14 |
| *Comparator* | 7 | 26 | 10 | 7 |
| Middle degree of problems |  |  |  |  |
| *Intervention* | 22 | 65 | 9 | 16 |
| *Comparator* | 20 | 61 | ? | 15 |
| High degree of problems |  |  |  |  |
| *Intervention* | 19 | 47 | 1518 |  |
| *Comparator* | 23 | 83 | 17 | 19 |
| *Only aggregated data were available to us. | | | | |

| **Supplemental table 2.** Missing values and for all variables used for the analyses in 2018 and 2021. | | | | |
| --- | --- | --- | --- | --- |
| Variables | Sample in 2018 | Missing values 2018 | Sample in 2021 | Missing values 2021 |
| **Sociodemographic variables** |  |  |  |  |
| Age | 6.0 (3.0-8.8) | 0 | 5.0 (2.0-9.0) | 1 |
| *0- to 4-year-olds* | 342 (34.8%) |  | 144 (34.9%) |  |
| *4- to 12-year-olds* | 642 (65.2%) |  | 268 (64.9%) |  |
| Gender |  | 0 |  | 1 |
| *Boy* | 492 50.0%) |  | 197 (47.7%) |  |
| *Girl* | 492 (50.0%) |  | 215 (52.1%) |  |
| **Socioeconomic status indicator** |  |  |  |  |
| Parental education |  |  |  |  |
| *High* | 504 (51.2%) |  | 238 (58.6%) |  |
| *Low* | 444 (45.1%) | 36 | 168 (41.4%) | 7 |
| **Outcomes in 0-to 12-year-olds** |  |  |  |  |
| Informal parenting support |  | 7 |  | 2 |
| *Yes* | 578 (58.7%) |  | 289 (70.3%) |  |
| *No* | 399 (40.5%) |  | 122 (29.7%) |  |
| Outdoor-play |  | 66 |  | 30 |
| *Yes* | 518 (52.6%) |  | 152 (39.7%) |  |
| *No* | 400 (40.7%) |  | 231 (60.3%) |  |
| General health |  | 5 |  | 0 |
| *Good* | 898 (91.3%) |  | 390 (94.4%) |  |
| *Not good* | 81 (8.2%) |  | 23 (5.6%) |  |
| **Outcomes in 4- to 12-year-olds** |  |  |  |  |
| Sport club membership |  | 0 |  | 0 |
| *Yes* | 354 (56.1%) |  | 145 (54.7%) |  |
| *No* | 277 (43.9%) |  | 120 (45.3%) |  |
| Risk of emotional and behavioural difficulties |  | 2 |  | 0 |
| *No* | 559 (88.9%) |  | 237 (89.4%) |  |
| *Yes* | 70 (11.1%) |  | 28 (10.6%) |  |
| MHP=mental health problems. Outcome measures 0-12- year olds sample size in 2018 N=984 sample size in 2021 N=413. Outcome measures 4-12-year olds sample size in 2018 N=631and sample size in 2021 N=265. Percentages are column percentages. P-values computed using chi-square for categorical variables and Mann Whitney U tests for continuous variables. | | | | |

| **Supplemental table 3.** Characteristics of the intervention and comparator neighbourhoods at baseline in 2018 and in 2021. | | | | | | |
| --- | --- | --- | --- | --- | --- | --- |
|  | Comparator neighbourhoods | | | Intervention neighbourhoods | | |
|  | 2018 (n=427; 71.5%) | 2021 (n=170; 28.5%) | p-value for change between T0 and T1 | 2018 (n=557; 69.6%) | 2021 (n=243; 30.4%) | p-value for change between T0 and T1 |
| **Socio-demographic variables** | | | | | | |
| Age, continuous | 6.0 (3.0-9.0) | 6.0 (2.8-9.0) | 0.783 | 5.0 (2.0 (8.0) | 5.0 (2.0-9.0) | 0.694 |
| Age, dichotomous |  |  | 0.370 |  |  | 0.488 |
| 0-4 | 132 (30.9%) | 59 (34.7%) |  | 210 (37.7%) | 85 (35.1%) |  |
| 4-12 | 295 (69.1%) | 111 (65.3%) |  | 347 (62.3%) | 157 (64.9%) |  |
| Age, categories |  |  | 0.345 |  |  | 0.705 |
| 0-4 | 132 (30.9%) | 59 (34.7%) |  | 210 (37.7%) | 85 (35.1%) |  |
| 4-10 | 224 (52.5%) | 78 (45.9%) |  | 254 (45.6%) | 118 (48.8%) |  |
| 10-12 | 71 (16.6%) | 33 (19.4%) |  | 93 (16.7%) | 39 (16.1%) |  |
| Gender |  |  | 0.559 |  |  | 0.637 |
| *Boy* | 216 (50.6%) | 81 (47.9%) |  | 276 (49.6%) | 116 (47.7%) |  |
| *Girl* | 211 (49.4%) | 88 (52.1%) |  | 281 (50.4%) | 127 (52.3%) |  |
| **SES** |  |  |  |  |  |  |
| Parental education |  |  | 0.165 |  |  | 0.198 |
| *Higher* | 228 (55.6%) | 104 (61.9%) |  | 276 (51.3%) | 134 (56.3%) |  |
| *Lower and intermediate* | 182 (44.4%) | 64 (38.1%) |  | 262 (48.7%) | 104 (43.7%) |  |
| **Outcomes 0- to -12-year-olds** | | | | | | |
| Informal parenting support |  |  | **0.002** |  |  | **0.012** |
| *Yes* | 238 (56.3%) | 118 (69.8%) |  | 340 (61.4%) | 171 (70.7%) |  |
| *No* | 185 (43.7%) | 51 (30.2%) |  | 214 (38.6%) | 71 (29.3%) |  |
| Outdoor-play |  |  | **0.021** |  |  | 0.872 |
| *Yes* | 168 (41.6%) | 49 (31.0%) |  | 232 (45.1%) | 103 (45.8%) |  |
| *No* | 236 (58.4%) | 109 (69.0%) |  | 282 (54.9%) | 122 (54.2%) |  |
| General health |  |  | 0.089 |  |  | 0.367 |
| *Good* | 389 (92.0%) | 163 (95.9%) |  | 509 (91.5%) | 227 (93.4%) |  |
| *Not Good* | 34 (8.0%) | 7 (4.1%) |  | 47 (8.5%) | 16 (6.6%) |  |
| **Outcomes in 4- to- 12-year-olds** | | | | | | |
| Sport club membership |  |  | 0.672 |  |  | 0.414 |
| *Yes* | 164 (56.7%) | 65 (59.1%) |  | 190 (55.6%) | 80 (51.6%) |  |
| *No* | 125 (43.3%) | 45 (40.9%) |  | 152 (44.4%) | 75 (48.4%) |  |
| Risk of emotional and behavioural difficulties |  |  | 0.259 |  |  | 0.580 |
| *No* | 257 (88.9%) | 102 (92.7%) |  | 302 (88.8%) | 135 (87.1%) |  |
| *Yes* | 32 (11.1%) | 9 (7.3%) |  | 38 (11.2%) | 20 (12.9%) |  |
| P-values computed using chi-square for categorical variables and Mann Whitney U tests for continuous variables.  **Bold** indicates a significant difference between T0 (2018) and T1 (2021). | | | | | | |

| **Supplemental table 4.** Exploratory analyses using difference-in-difference regression analyses. | | | | | | | |
| --- | --- | --- | --- | --- | --- | --- | --- |
|  | Emotional problems 4- to 12-year-olds | Anxiety 4- to 12-year-olds | Conduct problems 4- to 12-year-olds | Bullying 4- to 12-year-olds | Tantrums 4- to 12-year-olds | Stealing 4- to 12-year-olds | Being bullied 4- to 12-year-olds |
|  | OR (95%CI) N=896 | | | | | | |
| Model 1 | Two-way interaction parameter estimates (intervention condition in 2021) | | | | | | |
|  | 2.07 (0.70, 6.08) | 0.95 (0.48, 1.89) | 0.99 (0.47, 2.05) | 0.73 (0.26, 2.01) | 1.45 (0.78, 2.70) | 0.91 (0.39, 2.13) | 1..28 (0.60, 2.75) |
| Model 2 | Three-way interaction parameter estimates (difference in health inequalities for the intervention condition in 2021) | | | | | | |
|  | 1.29 (0.14, 11.71) | 1.47 (0.36, 5.96) | 0.94 (0.08, 11.32) | 0.58 (0.07, 4.83) | 0.92 (0.26, 3.31) | 2.41 (0.39, 14.74) | 0.34 (0.07, 1.61) |
| An odds ratio <1.00 indicates a favourable change in the outcome. **Bold** indicates statistical significance p <0.05. Model 1 is adjusted for age (continuous), gender (ref=boy) and parental education (ref=high), and includes a two-way interaction of time of measurement*condition. Model 2 is adjusted for age (continuous) gender (ref=boy) and parental education (ref=high) and includes two-way interactions of time of measurement*condition time of measurement*parental education condition*parental education and a three-way interaction of time of measurement*condition*parental education. | | | | | | | |

| **Supplemental table 5.** Difference-in-difference regression analyses in the complete-case dataset. | | | | | |
| --- | --- | --- | --- | --- | --- |
|  | Informal parenting support 0-to 12-year-olds | Outdoor-play 0- to 12-year-olds | General health 0- to 12-year-olds | Sport club membership 4- to 12-year-olds | Risk of emotional and behavioural difficulties  4- to 12-year-olds |
|  | OR (95% CI) for N=1,378 | OR (95% CI) for N=1,397 | OR (95% CI) for N=1,378 | OR (95%CI) for  N=896 | OR (95%CI) for  N=833 |
| Model 1 | Two-way interaction parameter estimates (intervention condition in 2021) | | | | |
|  | 1.11 (0.65, 1.88) | **0.58 (0.35, 0.97)** | 1.55 (0.52, 4.59) | 1.26 (0.67, 2.37) | 2.20 (0.77, 6.32) |
| Model 2 | Three-way interaction parameter estimates (difference in inequalities for the intervention condition in 2021) | | | | |
|  | **0.31 (0.11, 0.92)** | 0.93 (0.33, 2.64) | 1.04 (0.11, 9.90) | 0.59 (0.16, 2.15) | 0.99 (0.12, 8.40) |
| An odds ratio <1.00 indicates a favourable change in the outcome. **Bold** indicates statistical significance p <0.05. Model 1 is adjusted for age (continuous), gender (ref=boy) and parental education (ref=high), and includes a two-way interaction of time of measurement*condition. Model 2 is adjusted for age (continuous) gender (ref=boy) and parental education (ref=high) and includes two-way interactions of time of measurement*condition time of measurement*parental education condition*parental education and a three-way interaction of time of measurement*condition*parental education. | | | | | |

| **Supplemental table 6.** Difference-in-difference regression analyses on intermediate and ultimate outcomes with another control neighbourhood | | | | | |
| --- | --- | --- | --- | --- | --- |
|  | Informal parenting support 0- to 12-year-olds | Outdoor-play 0- to 12-year-olds | General health 0- to 12-year-olds | Sport club membership 4-to 12-year-olds | Risk of emotional and behavioural difficulties  4- to 12-year-olds |
|  | OR (95% CI) for N=1,397 | OR (95% CI) for N=1,397 | OR (95% CI) for N=1,397 | OR (95%CI) for  N=896 | OR (95%CI) for  N=896 |
| Model 1 | Two-way interaction parameter estimates (intervention condition in 2021) | | | | |
|  | 1.14 (0.68, 1.91) | **0.56 (0.34, 0.93)** | 1.58 (0.57, 4.38) | 1.53 (0.81, 2.86) | 2.40 (0.82, 7.03) |
| Model 2 | Three-way interaction parameter estimates (difference inequalities for the intervention condition in 2021) | | | | |
|  | 0.63 (0.22, 1.79) | 0.85 (0.30, 2.44) | 0.49 (0.06, 3.86) | 1.06 (0.29, 3.87) | 0.54 (0.06, 4.87) |
| An odds ratio <1.00 indicates a favourable change in the outcome. **Bold** indicates statistical significance p <0.05. Model 1 is adjusted for age (continuous), gender (ref=boy) and parental education (ref=high), and includes a two-way interaction of time of measurement*condition. Model 2 is adjusted for age (continuous) gender (ref=boy) and parental education (ref=high) and includes two-way interactions of time of measurement*condition time of measurement*parental education condition*parental education and a three-way interaction of time of measurement*condition*parental education. | | | | | |

| **Supplemental table 7.** Differences in SES between comparator and intervention neighbourhoods over time. | | | | | | | | | |
| --- | --- | --- | --- | --- | --- | --- | --- | --- | --- |
|  | 2018 | | | | | 2021 | | | |
|  | Comparator neighbourhoods | | Intervention neighbourhoods | | | Comparator neighbourhoods | | Intervention neighbourhoods | |
| %, N | Low SES | High SES | | Low SES | High SES | Low SES | High SES | Low SES | High SES |
| Age |  |  | |  |  |  |  |  |  |
| *0- to 4-year-olds* | 25.3% (46) | 36.8% (84) | | 30.2% (79) | 44.9% (124) | 25.0% (16) | 40.4% (42) | 26.0% (27) | 42.5% (57) |
| *4- to 12-year-olds* | 74.7% (135) | 63.2% (144) | | 69.8% (183) | 55.1% (152) | 75.0% (48) | 59.6% (62) | 74.0% (77) | 57.5% (77) |
| Gender |  |  | |  |  |  |  |  |  |
| *Boy* | 52.7% (96) | 47.4% (108) | | 48.9% (128) | 49.3% (136) | 47.6% (30) | 47.1% (49) | 51.9% (54) | 44.0% (59) |
| *Girl* | 47.3% (86) | 52.6% (120) | | 51.1% (134) | 50.7% (140) | 52.4% (33) | 52.9% (55) | 48.1% (50) | 56.0% (75) |
| **Outcomes in 0- to 12-year-olds** | | | | | | | | | |
| Informal parenting support |  |  | |  |  |  |  |  |  |
| *Yes* | 48.3% (87) | 63.6% (145) | | 54.8% (143) | 69.2% (191) | 54.7% (35) | 78.6% (81) | 68.9% (71) | 73.1% (98) |
| *No* | 51.7% (93) | 36.4% (83) | | 45.2% (118) | 30.8% (85) | 45.3% (29) | 21.4% (22) | 31.1% (32) | 26.9% (36) |
| Outdoor-play |  |  | |  |  |  |  |  |  |
| *Yes* | 38.6% (66) | 44.9% (96) | | 43.1% (106) | 47.2% (118) | 28.3% (17) | 31.3% (30) | 47.0% (47) | 45.5% (55) |
| *No* | 61.4% (105) | 55.6% (120) | | 56.9% (140) | 52.8% (132) | 71.7% (43) | 68.8% (66) | 53.0% (53) | 54.5% (66) |
| General health |  |  | |  |  |  |  |  |  |
| *Good* | 90.0% (162) | 94.2% (213) | | 87.0% (228) | 95.3% (262) | 95.3% (61) | 96.2% (100) | 92.3% (96) | 94.0% (126) |
| *Not Good* | 10.0% (18) | 5.0% (13) | | 13.0% (34) | 4.7% (13) | 4.7% (3) | 3.8% (4) | 7.7% (8) | 6.0% (8) |
| **Outcomes in 4- to 12-year-olds** | | | | | | | | | |
| Sport club membership |  |  | |  |  |  |  |  |  |
| *Yes* | 47.7% (63) | 66.2% (94) | | 48.3% (87) | 64.7% (97) | 40.4% (19) | 72.6% (45) | 43.4% (33) | 60.0% (45) |
| *No* | 52.3% (69) | 33.8% (48) | | 51.7% (93) | 35.3% (53) | 59.6% (28) | 27.4% (17) | 56.6% (43) | 40.0% (30) |
| Risk of emotional and behavioural difficulties |  |  | |  |  |  |  |  |  |
| *No* | 86.4% (114) | 93.7% (133) | | 87.2% (156) | 91.3% (137) | 91.5% (43) | 95.2% (59) | 85.5% (65) | 88.0% (66) |
| *Yes* | 13.6% (18) | 6.3% (9) | | 12.8% (23) | 8.7% (13) | 8.5% (4) | 4.8% (3) | 14.5% (11) | 12.0% (9) |
| SES is measured by highest parental education. | | | | | | | | | |
